# An overlooked link: venous thromboembolism in obstructive sleep apnea

**DOI:** 10.1093/sleep/zsaf390

**Published:** 2025-12-08

**Authors:** Alberto Garcia-Ortega, Silvia Ponce, Grace Oscullo

**Affiliations:** Respiratory Department, Doctor Peset University Hospital, Valencia, Spain; Respiratory Department, Foundation for the Promotion of Health and Biomedical Research in the Valencian Region (FISABIO), Valencia, Spain; Respiratory Department, Doctor Peset University Hospital, Valencia, Spain; Respiratory Department, Foundation for the Promotion of Health and Biomedical Research in the Valencian Region (FISABIO), Valencia, Spain; Respiratory Department, Hospital Universitario y Politécnico La Fe, Valencia, Spain; Respiratory Department, La Fe Health Research Institute (IISLaFe), Valencia, Spain

For decades, the cardiovascular and metabolic consequences of sleep-disordered breathing have been examined almost exclusively through an arterial perspective. Obstructive sleep apnea (OSA) has been conceptualized within the continuum of hypertension, arrhythmia, coronary disease, and stroke—its nightly cycles of intermittent hypoxia, sympathetic surges, typically interpreted as insults to the systemic arterial tree through oxidative stress, endothelial dysfunction, and heightened coagulability [1]. Yet the venous circulation, and particularly the risk of venous thromboembolism (VTE), encompassing pulmonary embolism (PE) and deep venous thrombosis (DVT), has received comparatively little attention. This gap is noteworthy given that VTE remains a major global health burden and that mechanistic parallels between OSA and thrombogenesis have long been suspected.

The nationwide, population-based analysis by Kim et al. directly addresses this gap and compels a reconsideration of this paradigm. Using Korea’s National Health Insurance database, the authors identified more than 133 000 adults with newly diagnosed OSA and matched them to over 600 000 controls through robust propensity-score techniques that accounted for demographics, lifestyle factors, and relevant comorbidities. Over 7.5 years of follow-up, individuals with OSA exhibited a substantially higher incidence of VTE (287.5 versus 205.0 events per 100 000 person-years), corresponding to a 68% higher risk. The association was stronger for PE than for DVT (82% and 54% higher risk, respectively), and remained remarkably consistent across age, sex, body-mass index, smoking status, and comorbidity subgroups. Importantly, results were unchanged after excluding individuals with cancer, a major confounder in VTE research [2].

This analysis, powered by a national dataset and strengthened by extensive adjustments, represents the most compelling epidemiologic evidence to date that OSA is not solely an arterial disorder but a systemic vascular disease that extends into the venous domain. It reinforces findings from smaller cohorts and case–control studies reporting two- to three-fold higher odds of PE or DVT among patients with OSA [3–5], and it aligns with physiologic investigations demonstrating elevations in fibrinogen, plasminogen-activator inhibitor-1, D-dimer, and markers of impaired fibrinolysis [6–8]. Earlier studies were limited by sample size or insufficient adjustment for sociodemographic and comorbid factors. Kim et al. overcome these limitations, establishing at the population level what mechanistic work has suggested for years.

The biological plausibility of this association is well-founded. Recurrent upper-airway obstruction triggers cycles of hypoxia and reoxygenation that generate oxidative stress, systemic inflammation, and sympathetic activation [7, 8]. Endothelial cells exposed to these oscillations up-regulate adhesion molecules and procoagulant pathways, while platelets show enhanced activation [9]. Together, these underlying mechanisms reproduce key etiopathogenic elements of Virchow’s triad (endothelial dysfunction and hypercoagulability) during sleep. In a field dominated by environmental and circumstantial triggers (e.g. surgery, cancer, major trauma, immobilization), identifying intrinsic biological drivers of VTE is particularly valuable, as they may reveal new therapeutic targets.

Yet the relationship between OSA and VTE appears to be more complex than a unidirectional link. In fact, emerging data suggest a bidirectional interaction between OSA and acute PE. Longitudinal studies have shown that sleep-disordered breathing can fluctuate from the acute to the stable phase of PE, suggesting that the embolic event itself may worsen nocturnal breathing disturbances, which then partially regress as hemodynamics normalize [10]. Moreover, nocturnal hypoxemia during the acute phase of PE has been identified as an independent predictor of adverse outcomes, including mortality and recurrence [11]. Hypoxemia, therefore, serves both as a trigger of thrombotic vulnerability in OSA and as a predictor of poor prognosis once a VTE has occurred. This dynamic interplay may contribute to recurrent or unresolved emboli that, in susceptible individuals, can evolve into chronic thromboembolic disease (CTED) or chronic thromboembolic pulmonary hypertension (CTEPH), thereby perpetuating abnormalities in ventilatory-vascular coupling [12].

Together, these insights support a unifying conceptual framework: intermittent hypoxia and sympathetic activation in OSA create a prothrombotic vascular milieu that increases the likelihood of VTE; once PE occurs, altered pulmonary hemodynamics and gas exchange may exacerbate nocturnal hypoxemia, further amplifying vascular and ventilatory instability. Recognizing this loop has meaningful implications for diagnosis, risk stratification, and long-term patient monitoring.

From a clinical standpoint, several implications arise. First, patients with moderate to severe OSA—particularly those with obesity, reduced mobility, or scheduled for surgery—should be considered a population at increased risk of VTE. Preventive strategies, including early mobilization, individualized thromboprophylaxis, and careful perioperative planning, warrant consideration. Conversely, patients presenting with unprovoked PE or DVT may benefit from targeted screening for OSA, using clinical sleep history or validated screening tools to assess OSA probability and to determine the need for a diagnostic sleep study. Identifying sleep-related hypoxemia may uncover a modifiable risk factor for recurrence or incomplete recovery [12].

Second, these findings prompt a reassessment of how OSA is classified within cardiovascular medicine. The conventional separation between arterial and venous diseases is increasingly artificial; the molecular mediators driving vascular injury, such as oxidative stress, inflammation, and endothelial activation, operate across both circulatory systems. Recognizing VTE as part of the vascular consequences of OSA would align clinical guidelines with evolving epidemiologic and mechanistic evidence.

Third, the question of reversibility deserves focused investigation. Continuous positive airway pressure (CPAP) therapy improves endothelial function, reduces platelet activation, and lowers levels of procoagulant factors [13]. Observational studies suggest that adequate CPAP adherence may reduce cardiovascular events. Yet no randomized trial has specifically examined whether CPAP reduces the incidence or recurrence of VTE or prevents progression to CTED or CTEPH. Given the magnitude and consistency of the association reported by Kim et al.*,* such trials are now essential to address this critical gap in knowledge. It is plausible that mitigating nocturnal hypoxemia could interrupt the hypoxia–inflammation–thrombosis cascade and reduce both acute and chronic VTE-related complications.

Finally, these insights resonate within the clinical challenge of persistent symptoms after acute PE. Many patients report persistent dyspnea, exercise limitation, or impaired quality of life despite appropriate anticoagulation. Whether unrecognized sleep-disordered breathing contributes to these residual symptoms remains an open question, but its potential overlap with post-PE syndrome is increasingly recognized. Comprehensive post-embolic assessment should therefore include evaluation of sleep quality and nocturnal oxygenation.

Looking forward, the field is well-positioned to integrate epidemiologic, mechanistic, and interventional research. Prospective cohorts with detailed assessment of hypoxic burden, biomarkers of endothelial injury and coagulation, and structured VTE follow-up are needed to clarify temporal and causal relationships. Multi-omic approaches may identify new molecular intermediaries linking hypoxia to thrombosis. Ultimately, randomized trials evaluating CPAP or other OSA therapies as strategies for VTE prevention will be essential to move beyond observational associations and establish causality.

By demonstrating that OSA independently increases the risk of VTE, Kim et al. provide compelling population-level evidence for a long-suspected relationship and invite a reframing of how sleep apnea is understood as a vascular disease. OSA should be recognized not only as a driver of arterial pathology but also as a contributor to acute VTE. Acknowledging and treating OSA may therefore extend beyond improving sleep and reducing blood pressure, as it may also help prevent the burden of venous thromboembolic disease that links nocturnal pathophysiology to vascular risk.

## Disclosure statement

*Financial disclosures:* The author declares that he has no financial relationships or interests relevant to this manuscript.

*Non-financial disclosures:* The author declares no non-financial conflicts of interest.
